# Supplementary material for: Is absorptive capacity the "panacea" for organizational development? A META analysis of absorptive capacity and firm performance from the perspective of constructivism
Source: PLoS One. 2023 Feb 24;18(2):e0282321. doi: 10.1371/journal.pone.0282321 (PMC9956603; doi:10.1371/journal.pone.0282321)
Supplement: S1 Appendix — (PDF) [file pone.0282321.s001.pdf]

META analysis for ACAP

Study name	Time point	Correlation	Lower limit	Upper limit	Z-Value	p-Value
Ben-Oz	2015.000	-0.373	-0.512	-0.213	-4.393	0.000
Encarnacion Garcia-Sanchez	2018.000	0.329	0.183	0.461	4.282	0.000
Chinho Lin	2015.000	0.109	-0.057	0.269	1.290	0.197
Xueyuan Liu	2018.000	0.577	0.493	0.650	10.911	0.000
WILLIAM J.WALES	2013.000	0.323	0.215	0.424	5.632	0.000
YI-YING CHANG	2012.000	0.240	0.098	0.373	3.266	0.001
Felipe Hernandez?Perlines	2016.000	0.730	0.630	0.806	9.696	0.000
Victor J. Garcia-Morales	2010.000	0.318	0.201	0.426	5.135	0.000
Garcia-Sanchez	2018.000	0.223	0.070	0.365	2.835	0.005
Shaker A. Zahra	2008.000	0.180	0.048	0.306	2.662	0.008
Mahir Pradana	2020.000	0.150	-0.018	0.309	1.750	0.080
Nikolaos Tzokas	2015.000	0.443	0.309	0.561	5.931	0.000
Rothaermel	2008.000	0.172	0.007	0.328	2.041	0.041
Latukha1	2018.000	0.068	-0.112	0.245	0.739	0.460
Shoham1	2017.000	0.350	0.196	0.487	4.293	0.000
YAPING GONG	2013.000	0.210	0.050	0.359	2.567	0.010
FERNHABER	2012.000	0.100	-0.034	0.231	1.461	0.144
Emine Kale	2019.000	0.470	0.351	0.574	6.966	0.000
Maria Teresa Bolivar-Ramos	2013.000	0.126	-0.030	0.275	1.581	0.114
Hefu Liu	2013.000	0.520	0.430	0.600	9.696	0.000
Rehman	2020.000	0.451	0.371	0.524	9.888	0.000
MATHEW HUGHES	2014.000	0.070	-0.095	0.231	0.830	0.407
Min Xue	2019.000	0.405	0.296	0.503	6.793	0.000
Imamoglu	2019.000	0.502	0.340	0.634	5.485	0.000
Felipe Hernandez-Perlines	2017.000	0.656	0.573	0.726	11.521	0.000
Jeong-Duk Choi	2017.000	-0.012	-0.149	0.126	-0.167	0.868
Sanjay Chaudhary	2018.000	0.110	-0.009	0.226	1.811	0.070
DURST	2018.000	0.378	0.206	0.527	4.127	0.000
Jung-Chieh Lee1	2016.000	0.546	0.398	0.666	6.271	0.000
ZUBIELQUI	2016.000	0.242	0.177	0.305	7.134	0.000
Iyengar	2015.000	0.110	0.040	0.179	3.085	0.002
PANKAJ C. PATEL	2014.000	0.208	0.047	0.357	2.527	0.012
Murad Ali	2016.000	0.350	0.220	0.468	5.064	0.000
Larraneta1	2017.000	-0.096	-0.258	0.071	-1.127	0.260
Rangus	2017.000	0.140	0.045	0.232	2.881	0.004
Fiona K. Yao	2017.000	0.105	-0.063	0.267	1.229	0.219
Lin Cui	2018.000	0.790	0.751	0.824	21.642	0.000
Molina	2018.000	0.439	0.339	0.529	7.853	0.000
Bapuji	2011.000	0.240	0.113	0.360	3.647	0.000
Aiqi Wu	2015.000	-0.036	-0.189	0.119	-0.454	0.650
LEUSCHNER	2015.000	0.134	0.060	0.208	3.507	0.000
Hughes	2018.000	0.460	0.324	0.578	6.030	0.000
Sciascia	2014.000	0.269	0.080	0.440	2.758	0.006
Engelen	2014.000	0.530	0.427	0.619	8.673	0.000
Jose Luis Ferreras-Mendez	2015.000	0.280	0.090	0.450	2.862	0.004
Kostopoulos	2011.000	0.095	0.004	0.185	2.039	0.041
Kohtamaki	2019.000	0.140	-0.074	0.342	1.284	0.199
Bouguerra	2021.000	0.410	0.289	0.518	6.191	0.000
Gerard George	2001.000	0.368	0.216	0.501	4.562	0.000
Raisal	2020.000	0.460	0.351	0.557	7.426	0.000
Seepana	2020.000	0.690	0.627	0.744	14.930	0.000
Feng Liu	2020.000	0.250	0.033	0.444	2.256	0.024
Kuen-Hung Tsai	2017.000	0.210	0.115	0.302	4.258	0.000
Jose Luis Ferreras?Mendez	2019.000	0.230	0.101	0.351	3.466	0.001
Imran Ali	2020.000	0.560	0.445	0.657	8.005	0.000
Zamora	2021.000	0.232	0.125	0.334	4.174	0.000
Ribin Seo	2021.000	0.371	0.250	0.480	5.712	0.000
Yu-Ming Zhai	2018.000	0.301	0.195	0.400	5.371	0.000
		0.309	0.247	0.368	9.370	0.000

Correlation and 95% CI

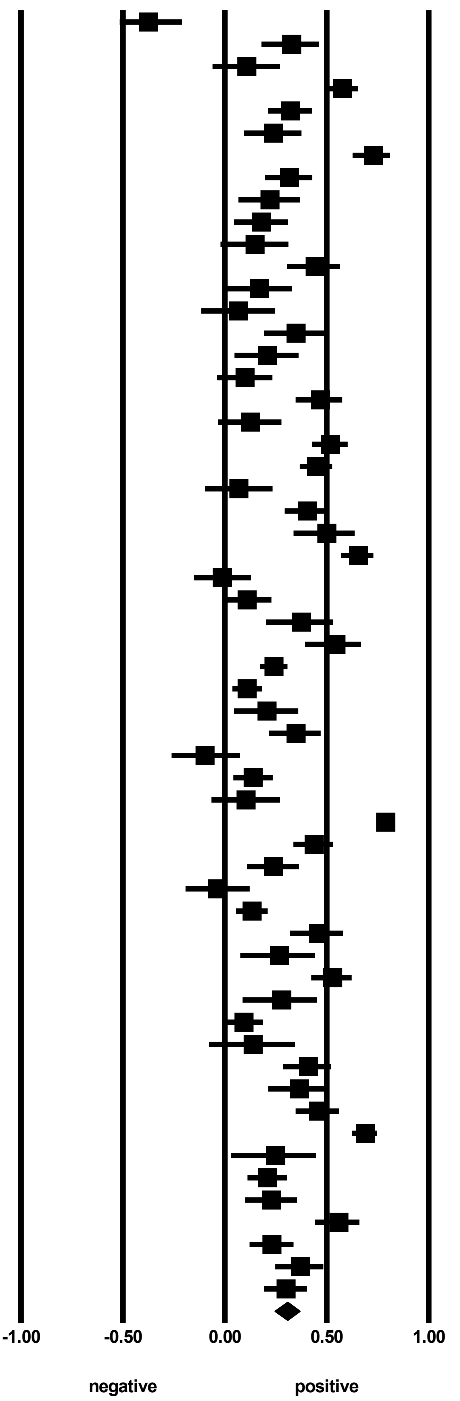

Meta Analysis
