# Supplementary material for: Is absorptive capacity the "panacea" for organizational development? A META analysis of absorptive capacity and firm performance from the perspective of constructivism
Source: PLoS One. 2023 Feb 24;18(2):e0282321. doi: 10.1371/journal.pone.0282321 (PMC9956603; doi:10.1371/journal.pone.0282321)
Supplement: S4 Appendix — (DOCX) [file pone.0282321.s004.docx]

**Identification of studies via databases and registers**

Records identified from*:

EBSCO、Web of Science、Elsevier ScienceDirect、SpringerLink、Wiley Online Library and ProQuest

Using searching formula：“absorptive capacity OR absorptive capacities OR ACAP OR ACAP capability AND performance OR firm performance OR financial performance OR organization performance”

**Identification**

Search result combined

(n = 316 )

Records excluded (n = 11 )

Duplications in databases

Records excluded(n =153 )

Absorptive capacity not as a main effect, moderating effect or mediating effect

Articles screened on basis of title and abstract

(n =305 )

**Screening**

Full-text articles assessed for eligibility

(n = 152)

Full-text excluded:

Not include quantitative measure:32

Do not have correlational matrix or measure that are convertible to a correlational effect size:62

Studies included in quantitative synthesis(meta-analysis

(n = 58)

**Included**

*Consider, if feasible to do so, reporting the number of records identified from each database or register searched (rather than the total number across all databases/registers).

**If automation tools were used, indicate how many records were excluded by a human and how many were excluded by automation tools.

*From:*  Page MJ, McKenzie JE, Bossuyt PM, Boutron I, Hoffmann TC, Mulrow CD, et al. The PRISMA 2020 statement: an updated guideline for reporting systematic reviews. BMJ 2021;372:n71. doi: 10.1136/bmj.n71

For more information, visit: <http://www.prisma-statement.org/>
