# Supplementary material for: Is absorptive capacity the "panacea" for organizational development? A META analysis of absorptive capacity and firm performance from the perspective of constructivism
Source: PLoS One. 2023 Feb 24;18(2):e0282321. doi: 10.1371/journal.pone.0282321 (PMC9956603; doi:10.1371/journal.pone.0282321)
Supplement: S5 Appendix — (DOCX) [file pone.0282321.s005.docx]

| Tile | Author | journal | No. |
| --- | --- | --- | --- |
| A Patent-Based Study Of The Relationships Among Technological Portfolio, Ambidextrous Innovation, And Firm Performance | Chinho Lin,  Chia-Chi Chang | Technology Analysis & Strategic Management |  |
| Absorptive Capacity And Business Performance The Mediating Effects Of Innovation And Mass Customization | Xueyuan Liu,  Haiyun Zhao | Industrial Management & Data Systems |  |
| Absorptive Capacity And Firm Performance: The Mediating Role Of Strategic Agility | Emine Kalea,  Ahmet Aknar | International Journal of Hospitality Management |  |
| Absorptive Capacity And Performance: The Role Of Customer Relationship And Technological Capabilities In High-Tech SMEs | Nikolaos Tzokasa, Young Ah. Kimb, Hammad Akbarc,  Haya Al-Dajanid | Industrial Marketing Management |  |
| Absorptive Capacity And Small Family Firm Performance: Exploring The Mediation Processes | Sanjay Chaudhary ,  Safal Batra | Journal Of Knowledge Management |  |
| Ambidexterity In Technology Sourcing: The Moderating Role Of Absorptive Capacity | Frank T. Rothaermel,  Maria Tereza Alexandre | Organization Science |  |
| Analysis Of The Influence Of The Environment, Stakeholder Integration Capability, Absorptive Capacity, And Technological Skills On Organizational Performance Through Corporate Entrepreneurship | Encarnacion GarcÍA-SÁNchez1 ,  VÍCtor JesÚS GarcÍA-Morales2 ,  Rodrigo MartÍN-Rojas2 | Int Entrep Manag J |  |
| Connecting External Knowledge Usage And Firm Performance: An Empirical Analysis | Hari Bapuji A,  David Loree B,  Mary Crossan | Journal Of Engineering And Technology Management |  |
| Core Knowledge Employee Creativity And Firm Performance: The Moderating Role Of Riskiness Orientation, Firm Size, And Realized Absorptive Capacity | Yaping Gong,  Jing Zhou,  Song Chang | Personnel Psychology |  |
| Depth And Breadth Of External Knowledge Search And Performance: The Mediating Role Of Absorptive Capacity | JosÉ Luis Ferreras-MÉNdez,  Sue Newell, Anabel FernÁNdez-Mesa, JoaquÍN Alegre | Industrial Marketing Management |  |
| Direct And Configurational Paths Of Absorptive Capacity And Organizational Innovation To Successful Organizational Performance | Murad Ali,  Konan Anderson Seny Kan B,  Marko Sarstedt | Journal Of Business Research |  |
| Do Individual Employees’ Learning Goal Orientation And Civic Virtue Matter? A Micro-Foundations Perspective On Firm Absorptive Capacity | Fiona K. Yao ,  Song Chang | Strategic Management Journal |  |
| Early Efforts To Develop Absorptive Capacity And Their Performance Implications: Differences Among Corporate And Independent Ventures | Ba´Rbara Larran˜eta1,  Jose´ Luis Gala´N Gonza´Lez2,  Rocio Aguilar | J Technol Transf |  |
| Effects Of Technology Absorptive Capacity And Technology Proactivity On Organizational Learning, Innovation And Performance: An Empirical Examination | VÍCtor J. GarcÍA-Morales ,  Antonia Ruiz-Moreno ,  Francisco Javier Llorens-Montes | Technology Analysis & Strategic Management |  |
| Entrepreneurial Orientation-As-Experimentation And Firm Performance: The Enabling Role Of Absorptive Capacity | Pankaj C. Patel,  Marko Kohtamäki,  Vinit Parida,  And Joakim Wincent | Strategic Management Journal |  |
| Entrepreneurial Orientation In Low- And Medium-Tech Industries: The Need For Absorptive Capacity To Increase Performance | Salvatore Sciascia A, Laura D’Oria B, Massimiliano Bruni A, BÁRbara Larrañeta | European Management Journal |  |
| Entrepreneurial Orientation In Turbulent Environments: The Moderating Role Of Absorptive Capacity | Andreas Engelena, Harald Kubea,  Susanne Schmidta, Tessa Christina Flattenb | Research Policy |  |
| Examining Absorptive Capacity In Supply Chains: Linking Responsive Strategy And Firm Performance | David D. Dobrzykowski, Rudolf Leuschner | Journal Of Supply Chain Management |  |
| Expatriate Knowledge Transfer, Subsidiary Absorptive Capacity, And Subsidiary Performance | Yi-Ying Chang,  Yapemg Gong,  Mike W. Peng | Academy Of Management Journal |  |
| Explaining The Entrepreneurial Orientation–Performance Relationship In Emerging Economies: The Intermediate Roles Of Absorptive Capacity And Improvisation | Paul Hughes,  Ian R. Hodgkinson, | Asia Pac J Manag |  |
| Explicating The Relationship Of Entrepreneurial Orientation And Firm Performance: Underlying Mechanisms In The Context Of An Emerging Market | Lin Cuia,  Di Fanb,  Feng Guo,  Yi Fana | Industrial Marketing Management |  |
| Family Firm Performance: The Influence Of Entrepreneurial Orientation And Absorptive Capacity | Felipe HernÁNdez-Perlines ,  Juan Moreno-GarcÍA ,  Benito YÁÑez-Araque | Psycholmark. |  |
| How Do Young Firms Manage Product Portfolio Complexity? The Role Of Absorptive Capacity And Ambidexterity | Stephanie A. Fernhaber,  Pankaj C. Patel2 | Strategic Management Journal |  |
| Impact Of Absorptive Capability On Software Process Improvement  And Firm Performance | Jung-Chieh Lee,  Wen-Chin Hsu,  Chung-Yang Chen | Inf Technol Manag |  |
| Influence Of Technological Assets On Organizational Performance Through Absorptive Capacity, Organizational Innovation And Internal Labour Flexibility | EncarnaciÓN GarcÍA-SÁNchez ,  VÍCtor J. GarcÍA-Morales ,  Rodrigo MartÍN-Rojas | Sustainability |  |
| Information Technology And Firm Performance: Mediation Role Of Absorptive Capacity And Corporate Entrepreneurship In Manufacturing SMEs | Nabeel Rehman,  Sadaf Razaq,  Ammara Farooq, Nayab Mufti Zohaib ,  Mohammad Nazri | Technology Analysis & Strategic Management |  |
| Information Technology Use As A Learning Mechanism: The Impact Of It Use On Knowledge Transfer Effectiveness, Absorptive Capacity, And Franchisee Performance | Kishen Iyengar,  Jeffrey R. Sweeney | Mis Quarterly |  |
| Innovation As The Key To Gain Performance From Absorptive Capacity And Human Capital | Mahir Pradana,  Ana PÉRez-Luño,  Maria Fuentes-Blasco | Technology Analysis & Strategic Management |  |
| Knowledge Absorptive Capacity, Innovation, And Firm’S Performance: Insights From The South Of Brazil | Guillermo Antonio Davila,  Susanne Durst | International Journal Of Innovation Management |  |
| Knowledge Inflows From Market- And Science-Based Actors, Absorptive Capacity, Innovation And Performance: A Study Of SMEs | Graciela Corral De Zubielqui,  Janice Jones,  Laurence Lester | International Journal Of Innovation Management |  |
| Learning Orientation And Absorptive Capacity As Determinants Of Innovativeness And Firm Performance | Salih Zeki Imamoglu, Huseyin Ince,  Hulya Turkcan,  Ersin Fidan | The European Proceedings Of Social & Behavioural Sciences |  |
| Marketing And Technological Absorptive Capacities: Environmental Antecedents And Performance Outcomes In High-Tech Firms | Aviv Shoham,  Yoel Asseraf,  Sara Lev,  Avi Fiegenbaum | Journal Of Business-To-Business Marketing |  |
| Performance Implications Of Organizational Ambidexterity Versus Specialization In Exploitation Or Exploration: The Role Of Absorptive Capacity | Miguel SolÍS-Molinaa,  Miguel HernÁNdez-Espallardob,  Augusto RodrÍGuez-Orejuelac | Journal Of Business Research |  |
| Short- And Long-Term Performance Feedback And Absorptive Capacity | Chanan Ben-Oz,  Henrich R. Greve | Journal Of Management |  |
| Social Capital And Learning Advantages: A Problem Of Absorptive Capacity | Mathew Hughes, Robert E. Morgan, Duane Ireland,  Paul Hughes | Strategic Entrepreneurship Journal |  |
| Strategic Orientations, Firm Performance And The Moderating Effect Of Absorptive Capacity | Manuel-Alejandro Ibarra-Cisneros,  Marıa Del Rosario Demuner-Flores,  Felipe Hernandez-Perlines | Journal Of Strategy And Management |  |
| Talent Management, Absorptive Capacity, And Firm Performance: Does It Work In China And Russia? | Marina Latukha,  Anna Veselova | Hum Resour Manage |  |
| The Effect Of International Venturing On Firm Performance: The Moderating Influence Of Absorptive Capacity | Shaker A. Zahra,  James C. Hayton | Journal Of Business Venturing |  |
| The Impact Of IT Capabilities On Firm Performance: The Mediating Roles Of Absorptive Capacity And Supply Chain Agility | Hefu Liu A,  Weiling Ke B,  Kwok Kee Wei, Zhongsheng Hua | Decision Support Systems |  |
| The Interplay Of Decentralization, Employee Involvement And Absorptive Capacity On Firms' Innovation And Business Performance | Kaja Rangus,  Alenka Slavec | Technological Forecasting & Social Change |  |
| The Penetration Of Green Innovation On Firm  Performance: Effects Of Absorptive Capacity And Managerial Environmental Concern | Min Xue,  Francis Boadu ,  Yu Xie | Sustainability |  |
| The Performance Effect Of Two Different Dimensions Of Absorptive Capacity And Moderating Role Of Holding-Cash | Jeong-Duk Choi ,  Ji-Hoon Park | Technology Analysis & Strategic Management |  |
| Too Much Of A Good Thing? AbsorptiveCapacity, Firm Performance, And The Moderating Role Of Entrepreneurial Orientation | William J. Wales,  Vinit Parida,  Pankaj C. Patel | Strategic Management Journal |  |
| Training And Business Performance: The Mediating Role Of Absorptive Capacities | Felipe HernÁNdez‑Perlines, Juan Moreno‑GarcÍ,  Benito YÁñez‑Araque | Springerplus |  |
| When Does Absorptive Capacity Matter For International Performance Of Firms? Evidence From China | Aiqi Wua,  Hinrich Voss | International Business Review |  |
| Performance Effects Of Entrepreneurial Orientation, Strategic Intent And Absorptive Capacity Within Coopetitive Relationships | Chandrasekararao Seepana ,  Fahian Anisul Huq | International Journal Of Operations & Production Management |  |
| Absorptive Capacity And Organizational Performance In An Emerging Market Context: Evidence From The Banking Industry In Turkey | Abderaouf Bouguerra,  Kamel Mellahi ,  Keith Glaister,  Arash Sadeghi,  Yama Temouri ,  Ekrem Tatoglu | Journal Of Business Research |  |
| Absorptive Capacity, Innovation, And Financial Performance | Konstantinos Kostopoulos, Alexandros Papalexandris, Margarita Papachroni,  George Ioannou | Journal Of Business Research |  |
| An Empirical Study On Entrepreneurial Orientation, Absorptive Capacity, And SMEs’ Innovation Performance: A Sustainable Perspective | Yu-Ming Zhai,  Wan-Qin Sun,  Sang-Bing Tsai,  Zhen Wang,  Yu Zhao,  And Quan Chen | Sustainability |  |
| Can Entrepreneurial Orientation Improve R&D Alliance Performance? An Absorptive Capacity Perspective | Ribin Seo,  Jakob Edler ,  Silvia Massin | R&D Management |  |
| From External Knowledge To Competitive Advantage: Absorptive Capacity, Firm Performance, And The Mediating Role Of Labour Productivity | Feng Liu,  Dev K. Dutta, Kwangtae Park | Technology Analysis & Strategic Management |  |
| How Absorptive Capacity Moderates The Value Of Firm Innovativeness In Turbulent Markets | Kuen-Hung Tsai,  Shu-Yi Yang | Canadian Journal Of Administrative Sciences |  |
| How International SME's Vicarious Learning May Improve Their Performance? The Role Of Absorptive Capacity, Strength Of Ties With Local SMEs, And Their Prior Success Experiences | Imran Alia,  Murad Alib, Mohammad Asif Salama,  Zeeshan Ahmed Bhattic,  Ghulam Ali Araind,  Muhammad Burhane | Industrial Marketing Management |  |
| Implications Of Strategic Flexibility In Small Firms: The Moderating Role Of Absorptive Capacity | Sanjay Chaudhary | South Asian Journal Of Business Studies |  |
| Moderating Effect Of Absorptive Capacity On The Entrepreneurial Orientation Of International Performance Of Family Businesses | Felipe Hernandez-Perlines | Journal Of Family Business Management |  |
| Moderating Effect Of Proactivity On Firm Absorptive Capacity And Performance: Empirical Evidence From Spanish Firms | Rafael Sancho-Zamora ,  Isidro Peña-GarcÍA , Santiago GutiÉRrez-Broncano,  Felipe HernÁNdez-Perlines | Mathematics |  |
| The Effects Of Alliance Portfolio Characteristics And Absorptive Capacity On Performance A Study Of Biotechnology Firms | Gerard Georgea, Shaker A. Zahrab, Kathleen K. Wheatleyc,  Raihan Khand | Journal Of High Technology Management Research |  |
| Journal Of High Technology  Management Research | Ismail Raisal,  Arun Kumar Tarofder,  Aboobucker Ilmudeen | World Journal Of Entrepreneurship, Management And Sustainable Development |  |
